# Use of imaging biomarkers to assess perfusion and glucose metabolism in the skeletal muscle of dystrophic mice

**DOI:** 10.1186/1471-2474-12-127

**Published:** 2011-06-04

**Authors:** Nabeel Ahmad, Ian Welch, Robert Grange, Jennifer Hadway, Savita Dhanvantari, David Hill, Ting-Yim Lee, Lisa M Hoffman

**Affiliations:** 1 Imaging Program, Lawson Health Research Institute, (268 Grosvenor St.), London (N6A 4V2), Canada; 2 Imaging Research Laboratories, Robarts Research Institute, (100 Perth Drive), London (N6A 5K8), Canada; 3 Medical Biophysics, The University of Western Ontario, (1151 Richmond Street), London (N6A 5B8), Canada; 4 Veterinary Services and Care, The University of Western Ontario, (1151 Richmond Street), London (N6A 5B8), Canada; 5 Virginia Polytechnic Institute and State University, (201 Burruss Hall), Blacksburg (24061), USA; 6 Radiology Department, London Health Sciences Centre, (800 Commissioners Road East), London (N6A 5W9), Canada; 7 Anatomy & Cell Biology, The University of Western Ontario, (1151 Richmond Street), London (N6A 5B8), Canada

## Abstract

**Background:**

Duchenne muscular dystrophy (DMD) is a severe neuromuscular disease that affects 1 in 3500 boys. The disease is characterized by progressive muscle degeneration that results from mutations in or loss of the cytoskeletal protein, dystrophin, from the glycoprotein membrane complex, thus increasing the susceptibility of contractile muscle to injury. To date, disease progression is typically assessed using invasive techniques such as muscle biopsies, and while there are recent reports of the use of magnetic resonance, ultrasound and optical imaging technologies to address the issue of disease progression and monitoring therapeutic intervention in dystrophic mice, our study aims to validate the use of imaging biomarkers (muscle perfusion and metabolism) in a longitudinal assessment of skeletal muscle degeneration/regeneration in two murine models of muscular dystrophy.

**Methods:**

Wild-type (w.t.) and dystrophic mice (weakly-affected mdx mice that are characterized by a point mutation in dystrophin; severely-affected mdx:utrn-/- (udx) mice that lack functional dystrophin and are null for utrophin) were exercised three times a week for 30 minutes. To follow the progression of DMD, accumulation of ^18 ^F-FDG, a measure of glucose metabolism, in both wild-type and affected mice was measured with a small animal PET scanner (GE eXplore Vista). To assess changes in blood flow and blood volume in the hind limb skeletal muscle, mice were injected intravenously with a CT contrast agent, and imaged with a small animal CT scanner (GE eXplore Ultra).

**Results:**

In hind limb skeletal muscle of both weakly-affected mdx mice and in severely-affected udx mice, we demonstrate an early, transient increase in both ^18^F-FDG uptake, and in blood flow and blood volume. Histological analysis of H&E-stained tissue collected from parallel littermates demonstrates the presence of both inflammatory infiltrate and centrally-located nuclei, a classic hallmark of myofibrillar regeneration. In both groups of affected mice, the early transient response was succeeded by a progressive decline in muscle perfusion and metabolism; this was also evidenced histologically.

**Conclusions:**

The present study demonstrates the utility of non-invasive imaging biomarkers in characterizing muscle degeneration/regeneration in murine models of DMD. These techniques may now provide a promising alternative for assessing both disease progression and the efficacy of new therapeutic treatments in patients.

## Background

Duchenne muscular dystrophy (DMD) is a severe recessive X-linked neuromuscular disease that affects 1 in 3500 live-male births. The disease is characterized by progressive skeletal muscle degeneration that arises due to mutations in or loss of dystrophin from the dystrophin-glycoprotein complex (DGC) within the sarcolemmal membrane [1,2]. Dystrophin deficiency destabilizes the sarcolemma, and not only renders myofibers susceptible to contraction-induced damage [1,2], but also allows cytosolic Ca^2+ ^levels to increase, and initiates a cascade of intracellular events that lead to necrosis [3,4]. Skeletal muscle cell signaling is further affected through the mislocalization of neuronal nitric oxide synthase (nNOS) that is normally localized to the sarcolemma as part of the DGC [5-7]. Typically, this DGC protein plays a role in myofiber differentiation [8], modulation of contractile force [9], and exercise-induced glucose uptake [10]. In DMD, however, nNOS mislocalization has been shown to produce ischemia that, in conjunction with membrane leakage and necrosis, triggers an inflammatory reaction [11]. Histological analyses reveal necrotic or degenerating myofibers that are surrounded by macrophages and CD4+ lymphocytes [11].

While a diagnosis of DMD is usually made in patients approximately 5 years of age by assessment of family history and PCR genotyping [12], analysis of disease progression typically relies upon the measurement of muscle strength [13], creatine kinase levels [14] and painful, invasive muscle biopsies [15]. Previous studies have reported the use of magnetic resonance (MR) imaging to detect changes in muscle architecture [16] or sarcolemmal permeability [17] in dystrophic mice. Positron emission tomography (PET) has also been used to measure metabolic differences in myocardia of healthy wild-type (w.t.) versus mdx mice [18]. To date, however, there is no effective method to reliably and non-invasively assess degenerative changes that occur as DMD progresses, particularly the ischemic, metabolic and inflammatory changes that occur as a result of aberrant sarcolemmal signaling. There is also a clear unmet medical need to establish accurate monitoring of cell-based and other therapies, including exercise, for the treatment of muscle disease. Therefore, in the present study, we demonstrate the utility of dynamic contrast enhanced computed tomography (DCE-CT) and PET scanning to, firstly, longitudinally assess changes in muscle perfusion and metabolism as disease progresses in two murine models of DMD, and to, secondly, provide a baseline for monitoring the success (or failure) of various therapeutics.

## Methods

### Animal Models of Duchenne Muscular Dystrophy

Male wild-type (C57BL/6) and mdx (point mutation in dystrophin) mice were purchased from Charles River and Jackson Laboratories, respectively. Mdx:utrn heterozygote mice (generously provided by Robert Grange, Virginia Polytechnic Institute and State University, although originally generated by Mark Grady and Josh Sanes at Washington University, St. Louis,) were bred to generate mdx: utrn-/- (udx) mice lacking both utrophin and functional dystrophin. Only male mice were used in this study. Four groups of mice were used for imaging: 1) control wild-type (w.t. n = 9), which were exercised involuntarily, as described below; 2) exercised mdx (n = 9); 3) non-exercised mdx (n = 9); and 4) non-exercised udx (n = 11). The same four groups consisting of three mice in each were used for histology at time points specified below. All study procedures were approved by our institutional Animal Ethics Committee and conducted according to guidelines set by the Canadian Council on Animal Care.

### Exercise and Non-exercise protocol

Mdx and w.t. mice in the exercised group ran 3 times weekly for 30 minutes each time on a motorized treadmill (Accuscan Instruments), at a speed of 15 meters per minute (mpm) and a 7-degree uphill incline [19]. This regime was initiated when mice were 6 weeks of age, and continued until animals were 22 weeks of age. Due to the severity of their disease state, udx mice were unable to tolerate the exercise protocol, and as such, no exercise group was employed. A non-exercised w.t. group was also not employed in this study because our experimental design was to investigate the effect of chronic involuntary exercise on muscle degeneration/regeneration in dystrophic mouse models. To ensure that all animals in the study were handled similarly, non-exercised mdx and udx mice were walked on the treadmill 3 times weekly, for 10 minutes each time at 5 - 7mpm with no incline. Exercised animals began running 45 minutes prior to scans, and all groups allowed 15 minutes of rest before anesthetic induction. An additional 10-15 minutes was required for placement of a tail vein catheter for delivery of both contrast for DCE-CT scanning and for radiolabeled glucose substrate for PET scanning.

### DCE-CT and PET Imaging

**Figure 1:**
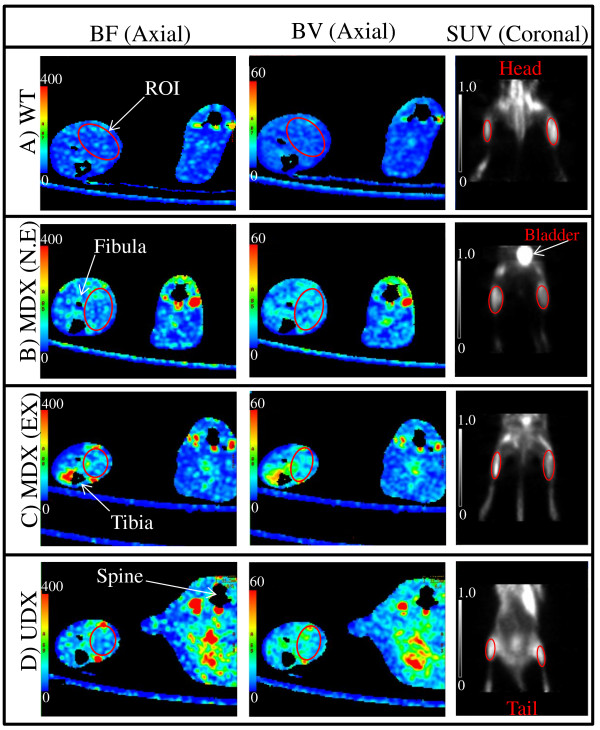
**DCE-CT image (a), Blood flow (b), Blood volume (c) and FDG-PET image (d) of a mdx mouse at 8 weeks of age**. Axial slices using DCE-CT of the lower limbs of a dystrophic mouse **(a,b,c); **coronal slice generated using PET, displaying the mdx mouse from the abdomen to the feet **(d)**. Red circles indicate one region of interest (ROI) encompassing the posterior compartment of the hind limb (HL) musculature from which data was collected.

To obtain baseline values, all mice were imaged using DCE-CT and PET at 6 weeks of age, prior to the initiation of either the exercise or non-exercise protocol. The mice were thereafter imaged biweekly for another 16 weeks up to 22 weeks of age. During each imaging session, anesthesia was induced with 3-4% isofluorane and then maintained with a 1.5% oxygen-balanced isofluorane mixture, delivered at a constant rate of 1L/min. All imaging attempted to focus on the gastrocnemius muscle (GM) of the hind limb since the size of the GM relative to other skeletal muscles in the posterior compartment of the hind limb (HL) allows for greater visibilility in longitudinal DCE-CT and PET images, and thus more consistent placement of regions of interest (ROIs); realistically, placement of ROIs included several muscles within the posterior compartment of HL musculature (Figure 1). Data acquired for each mouse over the course of the study were normalized with respect to baseline values for each mouse to minimize biological variability between different mice, thus allowing us to assess longitudinal changes between different groups of mice.

### (a)DCE-CT Scanning for Measurement of Muscle Blood Flow and Blood Volume

A 4 cm thick slab of both hind limbs, including the GMs, separated into forty 0.9 mm thick slices at 175 µm resolution, was scanned repetitively with a GE Healthcare eXplore Locus Ultra µCT scanner. The 5 minute scan duration was divided into two phases: first, 1 s scan for 30 s and then one 1 s scan every 15 s for the remainder of the scan duration. Five seconds into the first phase of scanning, contrast agent (150 μL of diluted Hypaque 300, 200 mg of iodine per ml) was injected via a tail vein catheter at an injection rate of 2.0 ml/min with an infusion pump (New Era Pump Systems Inc) that was triggered by the CT scanner. We used CT Perfusion software (GE Healthcare) to describe the exchange of contrast between the blood stream and the interstitial space to quantify blood flow (BF) and blood volume (BV) [20,21], and to calculate functional maps of these parameters from the acquired series of CT images [21] in healthy versus degenerating/regenerating muscle [22]. The tracer kinetics model used to calculate BF and BV maps is described in full in the Appendix. From the derived BF and BV maps, mean BF and BV in the HL musculature of a CT slice were derived by ROI analysis (Figure 1). Values from all slices of a mouse were weighted according to area and averaged, and the averaged values normalized by the corresponding baseline values. Results from mice respective to their own group and week were then averaged.

### (b)PET Scanning for Measurement of Glucose Metabolism

A small dose of a glucose analog, fluorine-18 labeled fluorodeoxyglucose (^18^F-FDG, 11-38 MBq), was administered via a tail vein catheter. Forty to sixty minutes after injection, HL musculature were imaged with a small animal PET scanner (GE Healthcare, eXplore Vista DR), using a photopeak window of 250-700 keV for a duration of 30 minutes. The acquired data were corrected for scatter and reconstructed using an OSEM algorithm into twenty-six, 1.7 mm thick slices at a transaxial resolution of 1.6 mm. The (sensitivity) factor for converting counts in reconstructed PET images to activity (in MBq) was determined at regular intervals by scanning with the PET scanner a 5 cm diameter water phantom filled with a known amount of F-18 activity (11-14 MBq). ROIs were drawn in coronal PET slices to encompass the posterior HL skeletal muscle compartment including the GM (Figure 1). Values from all slices of a mouse were weighted according to area and averaged. The average count for the HL muscle was converted into mean activity per ml using the conversion factor determined above, then normalized by the injected activity and the body weight of the animal to arrive at the standardized uptake value (SUV), a semi-quantitative indicator of the average glucose metabolic rate over the period from injection to measurement [23,24]. The SUVs were normalized by the baseline value for each mouse, and the normalized values then averaged respective to week and group.

### Histological analysis

Twelve extra mice were added to each of the above-mentioned 4 groups of mice, for sacrifice at baseline (6 weeks) and at 8, 14, and 22 weeks of age. Mice used for histology were handled and exercised in the same manner as imaged mice. For udx mice that did not survive up to 22 weeks of age, the GMs were harvested for histology at the time of their death, typically between 20-22 weeks of age. Following harvest, GMs were fixed in 10% neutral-buffered formalin for a minimum of 48 hours. The fixed muscle was then embedded in paraffin wax and cut transversely in 5-micron sections for subsequent staining with haematoxylin and eosin (H&E) [25]. Stained sections were evaluated for histological features characteristic of DMD, including fiber degeneration, fiber regeneration, hypercontracted fibers, myophagia, inflammatory infiltrate, fibrosis and necrosis. Whereas eccentric nuclei are typical of healthy myofibers, fibers that contain centralized nuclei are evidence of a regenerative process, thus reflecting a state in which resident muscle satellite cells differentiate into mature myocytes that engraft within damaged myofibers to initiate repair [26,27].

### Data analysis

Statistical analyses were performed using the SPSS Statistics software package for Windows (SPSS Inc., V15.0). Repeated measures ANOVA was used as an omnibus test to identify significant differences in normalized BF and BV, and SUV of ^18^F-FDG in the HL musculature among groups and time points within groups. Tukey tests were used for the post-hoc analyses of differences.

## Results

### Survival of mice in experimental groups

**Figure 2:**
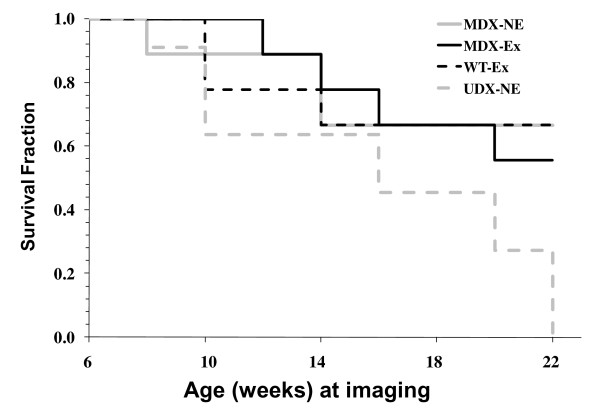
**Kaplan Meier survival curve of mice used in study**. Four experimental groups of mice were used in the longitudinal study after initiation of exercise regime: exercised wild-type (W.T.-Ex), non-exercised mdx (MDX-N.E.), exercised mdx (MDX-Ex) and exercised udx (UDX-Ex). Mice sacrificed at specified histological time points are not included in Kaplan Meier survival curve.

Kaplan Meier survival curves illustrate that there were significant differences in the survival rates of severely-affected udx mice relative to mice in each of the other three experimental groups (Figure 2). Specifically, 67%, 67%, 56% and 0% of mice in the exercised wild-type (w.t.), non-exercised mdx, exercised mdx and non-exercised udx groups, respectively, survived until the termination of the study.

### Early changes in perfusion, metabolism and histology following exercise

**Figure 3:**
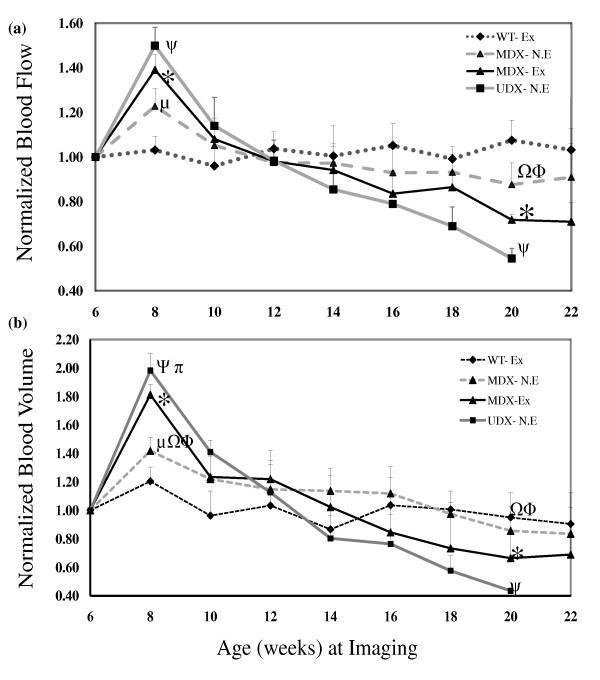
**Normalized blood flow, NBF (a) and normalized blood volume, NBV (b) in hind limb muscle (HL)**. Data was collected over a 14-16 week period from age 6 to 20-22 in exercised w.t. (W.T.-Ex), non-exercised mdx (MDX-N.E.), exercised mdx (MDX-Ex) and non-exercised udx (UDX-N.E.) mice. Plotted values are means and standard deviations of NBF and NBV of surviving mice from each group. Significant differences (p <0.05) were found at age 8 and 20 between: W.T.-Ex and MDX-N.E. (μ), W.T.-Ex and MDX-Ex (*), W.T.-Ex and UDX-N.E.(ψ), MDX-N.E. and UDX-N.E. (≏), MDX-N.E. and MDX-Ex (ϕ).

Absolute values of muscle perfusion (BF and BV), and glucose metabolic rate (SUV) measured at baseline for w.t., non-exercised mdx, exercised mdx and udx mice are provided in Table 1. As illustrated in Figure 3, normalized blood flow (NBF) and blood volume (NBV) in the HL musculature of healthy exercised w.t. mice did not change significantly from the initiation of the study at baseline to its termination. In each of the other 3 experimental groups, however, both NBF and NBV peaked 2 weeks post-baseline (8 weeks of age); specifically, mice in the non-exercised mdx group, the exercised mdx group, and the udx group exhibited an 18%, 38% and 42% increase in NBF above baseline, respectively (P < 0.05). Each experimental group exhibited similar changes in NBV, with a 40%, 79% and 97% increase above baseline observed in non-exercised mdx, exercised mdx and udx mice, respectively (P < 0.05). Significant differences in NBF and NBV were also observed between both mdx groups and the udx group, between non-exercised mdx and their exercised litter mates, and between all 3 groups and w.t. mice (P < 0.05).

**Table 1:** Absolute values (mean ± SD) of blood flow (BF), blood volume (BV), and standardized uptake value (SUV) for each experimental group at baseline

	Blood Flow (BF)	Blood Volume (BV)	Standardized Uptake Value (SUV)
**Wild-type (w.t.)**	33.85 ± 4.13	2.73 ± 0.49	0.38 ± 0.09
**MDX (N.E)**	42.33 ± 4.42	3.39 ± 0.48	0.36+0.08
**MDX (Ex)**	46.14 ± 4.27	3.54 ± 0.67	0.39 ± 0.1
**UDX **	54 ± 3.36	4.79 ± 0.55	0.46 ± 0.12

**Figure 4:**
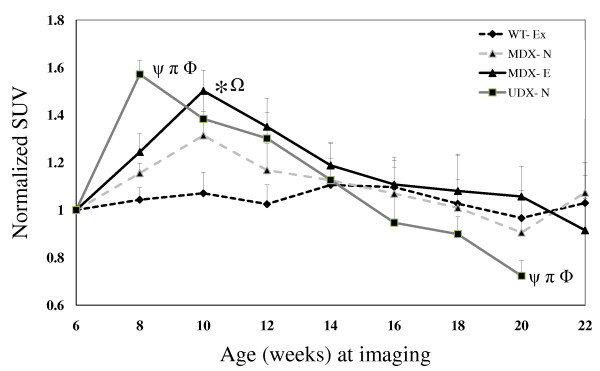
**Normalized SUVs (NSUV) of ^18^F-FDG in hind limb muscle (HL)**. Data was collected over a 14-16 week period from age 6 to 20-22 in exercised w.t. (W.T.-Ex), non-exercised mdx (MDX-N.E.), exercised mdx (MDX-Ex) and non-exercised udx (UDX-N.E.) mice. Plotted values are means and standard deviations of normalized SUV of surviving mice 23 from each group. Significant differences (p<0.05) are observed at week 8 and 20 between: W.T.-Ex and UDX –N.E. (ψ), MDX-Ex and UDX-N.E.(π), and MDX-N.E and UDX-N.E. (_); and, at week 4 between: W.T.-Ex and MDX-Ex (*), and MDX-N.E. and MDX-Ex (φ).

Analysis of glucose uptake, as assessed by normalized ^18^F-FDG SUV (NSUV) in the HL musculature of mice in each of the four experimental groups of mice revealed patterns similar to those observed for NBF and NBV (Figure 4). Specifically, at 4 weeks post-baseline (10 weeks of age), NSUVs in non-exercised and exercised mdx mice were higher than that observed in w.t. mice (P < 0.05), exhibiting 31% and 50% increases above baseline values, respectively. Severely-affected udx mice, in contrast, exhibited a pronounced peak in NSUV two weeks post-baseline. This value is higher than that of the w.t. mice (P <0.05) and represents a 62% increase above its baseline (P <0.05).

**Figure 5:**
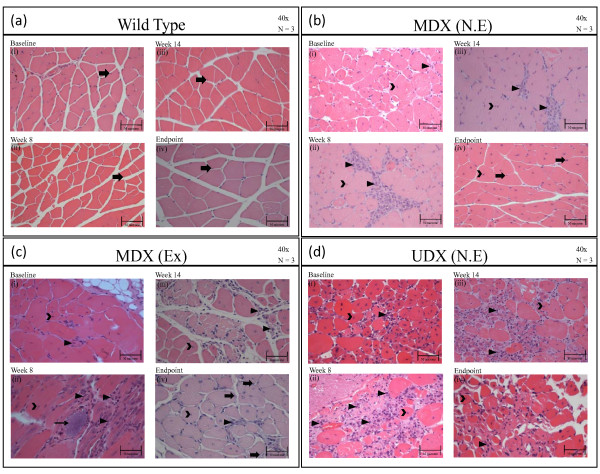
**H&E-stained sections of gastrocnemius muscles (GMs)**. H&E-stained sections of gastrocnemius muscles (GMs) isolated from: **(a) **wild-type (w.t.) mice at (i) baseline (6 weeks old), (ii) week 8, (iii) week 14, and (iv) at the termination of the study (20-22 weeks of age). Pathology is described in the text. Legend: solid arrow, points to eccentrically-located nuclei in myofibers; **(b) **non-exercised mdx at (i) baseline, (ii) week 8, (iii) week 14, and (iv) at the termination of the study. Legend: open arrowhead points to centrally-located nuclei in myofibers; closed arrowhead points to inflammatory infiltrate, solid arrow points to eccentrically-located nuclei in myofibers; **(c) **exercised mdx mice at (i) baseline, (ii) week 8, (iii) week 14, and (iv) at the termination of the study. The pathology observed in each group and at each time are described in the text. Legend: solid arrow points to eccentrically-located nuclei; open arrowhead points to centrally-located nuclei in myofibers; closed arrowhead points to inflammatory infiltrate; and thin solid arrow points to a mineralized lesion; (d) non-exercise udx mice at (i) baseline, (ii) week 8, (iii) week 14, and (iv) at the termination of the study. Legend: open arrowhead points to centrally-located nuclei in myofibers; closed arrowhead points to inflammatory infiltrate.

Analysis of H&E-stained GM tissue sections revealed that, at baseline (6 weeks of age) and at all time points thereafter, the GMs of w.t. mice were predominantly comprised of eccentrically-located nuclei (arrow), were devoid of inflammatory cells, and displayed normal amounts of connective tissue amongst myofibers (Figure 5a). In contrast, the GMs harvested from either group of mdx mice (non-exercised and exercised) at baseline exhibited numerous centrally-located nuclei (open arrow head), and small amounts of inflammatory infiltrate (arrow head) in the interstitial space (Figure 5b &5c(i)). The severity of the degenerative process in udx mice, relative to that occurring in mildly-affected mdx mice, was evidenced by the predominance of centrally-located nuclei (open arrow head) and the widespread presence of inflammatory infiltrate within enlarged intercellular space (arrow head) (Figure 5d(i)). Two weeks post-baseline (8 weeks of age), non-exercised mdx mice still exhibited centrally-located nuclei (open arrow head), as well as modest inflammatory infiltrate (arrow head) (Figure 5b(ii)). Centrally-located nuclei were also observed in exercised mdx and udx mice (open arrow head), although most notable in both groups was the presence of a marked inflammatory infiltrate amongst myofibers (arrow head) (Figure 5c and 5d(ii)), and by the occasional appearance of mineralized tissue (Figure 5c(ii), thin arrow).

### Intermediate and Late changes in perfusion, metabolism and histology following exercise

In healthy exercised w.t. mice, NBF, NBV and NSUV in the HL musculature did not change significantly over the duration of the study (Figure 3 & Figure 4). In the HL musculature of non-exercised mdx mice, however, a progressive decline in all three imaging biomarkers was observed: NBF (Figure 3a), NBV (Figure 3b) and NSUV (Figure 4), relative to their respective peak values at 8 weeks of age. Similar, albeit greater, declines in all three imaging biomarkers were observed in both exercised mdx mice and non-exercised udx mice. Importantly, these declines appear to reflect the disease state of each model, with mildly-affected mdx mice being intermediate to healthy w.t. mice and severely-affected udx mice; as expected, a greater decline is observed in exercised mdx mice relative to their non-exercised litter mates.

As illustrated in Figure 5, analysis of H&E-stained sections of GMs harvested from either non-exercised or exercised mdx mice at their endpoint revealed that many myofibers now exhibit a healthy wild-type phenotype; specifically, eccentrically-located nuclei, rather than centralized nuclei, predominate (open arrow head in Figure 5b and 5c(iii & iv)). As further evidence of this, by 22 weeks of age, there was only a mild inflammatory infiltrate in the exercised group and no visible inflammatory infiltrate in the non-exercised group. In udx mice, in comparison, myofibers exhibited advanced atrophy with ongoing myofibrillar degeneration, and mixed with marked inflammatory infiltrate (Figure 5d (iii & iv).

## Discussion

Muscle degeneration in DMD occurs due to mutations in or a loss of dystrophin [1,2] from the sarcolemmal glycoprotein membrane complex, and results in muscle fragility, contraction-induced damage, necrosis, and inflammation [3,4,11]. Inflammatory and immune processes are now believed to be particularly important during early disease progression, perhaps being initiated via aberrant signaling in dystrophic sarcolemma [28-35]. However, while cellular events such as these are typically analyzed histologically, their use as effective indices to monitor the *progression *of myopathy in dystrophic muscle is limited by an inability to longitudinally assess them, and in a non-invasive manner. In the present study, we conducted DCE-CT and PET scanning to demonstrate the utility of non-invasive imaging biomarkers to longitudinally assess muscle regeneration/degeneration in two murine models of DMD.

Over the years, there has been some debate as to the validity of using various murine models for studies of DMD. To date, the mdx mouse model is the best characterized, with gene expression profiles overlapping considerably with humans [36]. More recent studies have further shown that, as in DMD patients, both sarcolemmal stabilization and contractile mechanisms are impaired in myofibers isolated from mdx mice [37]. As there are many molecular aspects common to both the mdx mouse and DMD patients, there is a strong rationale for the use of the mdx mouse model in DMD studies. There are, however, important differences between the mdx mouse model and patients [38,39]; indeed initial disease onset in *mdx *mice occurs at ~3 weeks of age [40], The process of degeneration in *mdx *skeletal muscles wanes shortly thereafter, by ~ 4 weeks, at which time muscle regeneration predominates [40]. These cycles of muscle degeneration/regeneration are suppressed by ~ age 3-4 months, but continues at low levels for the remainder of the mouse's lifetime [40], thereby producing the mild phenotype well-associated with the mdx model. Importantly, the imaging of biomarkers conducted in the present study substantiates these reports. The weak phenotype in mdx mice is believed to be attributed, at least in part, to an upregulation of utrophin (a homolog of dystrophin) in the sarcolemmal DGC, and compensatory mechanisms for the loss of functional dystrophin [41]. Thus, the mdx:utrn-/- (udx) mouse model, in comparison, exhibits a far greater degree of muscle pathogenesis than that observed in mdx mice, closely paralleling the human condition [42]. Indeed, severely affected udx mice in our study exhibited progressive myofiber degeneration, with little repair throughout the duration of the study.

Previous studies have reported the use of enforced treadmill running to exacerbate muscle pathogenesis in mdx mice [19]. Exercise-induced myofiber damage has been further correlated with decreased expression of hormones such as IGF-1 and myogenic transcription factors such as Myo-D, which, in turn, contribute to the degenerative state [43]. More recently, several studies have concluded that *voluntary *exercise may be, in fact, be beneficial [44-47]. In the present study, we used an involuntary treadmill running protocol, although the regime was not eccentric; as such, we were uncertain whether we would indeed exacerbate muscle pathogenesis or slow disease progression. We observed little difference histologically between mdx mice exercised via involuntary, uphill running and their non-exercised littermates; at baseline to 2 weeks-post we observed, in both groups, the presence of numerous centrally-located nuclei and inflammatory infiltrate. Subsequent staining from 8 weeks-post through to the termination of the study demonstrated, in both exercised and non-exercised mdx mice, the presence of eccentrically-located nuclei and indicating that once-damaged muscle was reverting to a healthy wild-type phenotype. Traditional histology, however, tends to suffer from a sampling issue, as only a small fraction of tissue is analyzed but provides extremely high spatial resolution. In comparison, *in vivo *functional imaging, as presented in this study, permits measurement of muscle perfusion, blood volume and metabolic rate over the entire muscle mass, thus avoiding the above-mentioned sampling problem. We did, in fact, demonstrate that the uphill running regime used in our study was sufficient to induce changes in blood flow, blood volume and glucose metabolism, and that these changes appear to correlate with the severity of the disease state in each group of mice. More specifically, we observed a *transient increase *in blood flow, blood volume, and glucose uptake in mdx mice 2 weeks-post (both exercised and unexercised), relative to healthy wild-type control mice. Furthermore, we were now able to distinguish between exercised mdx and non-exercised mdx mice, with greater increases in blood flow, blood volume and glucose metabolism being exhibited by the former group. We suggest that these transient increases in perfusion and metabolism in both non-exercised and exercised mdx groups reflect active cycles of degeneration/regeneration and a moderate exacerbation of muscle pathogenesis, respectively. We believe that the greatest increase in blood flow, blood volume and glucose metabolism observed in udx mice is likely due to the severity of pathogenesis and inflammation in these mice. These conclusions were supported by histological findings. Given reports that cycles of degeneration/regeneration wane at ~ 3-4 months of age in mdx mice [40], we were not surprised to have observed a subsequent progressive decrease in blood flow, blood volume and metabolism. Importantly, our imaging of biomarkers enabled us in the present study to detect differences between exercised mdx mice and their non-exercised littermates, with the former exhibiting a greater decline in each biomarker. Not surprisingly, still greater declines in all imaging biomarkers was observed in severely-affected udx mice, findings that were, again, supported by our histological findings. To the best of our knowledge, there is no satisfactory method, to date, to account for the vastly different scales of resolution and sampling between traditional histology and *in vivo *functional imaging when the two types of results are compared. Nevertheless, we are confident that the qualitative histological findings illustrated in this study do indeed support our hemodynamic and metabolic measurements as functional imaging methods for the assessment of physiologic changes in degenerating/regenerating muscle *in vivo*.

There are several limitations in our study. Firstly, in each DCE-CT study, an approximate 26 mSv effective dose was administered to individual mice. Since wild-type mice did not exhibit overt muscle damage, primary radiation damage in dystrophic mice can be ruled out. However, it is possible that the radiation administered may have compounded ischemic and inflammatory effects to exacerbate damage observed in both mdx and udx mouse models. A second limitation of our study results from the exercise protocol; exercised animals were included to assess the effects of chronic damage in the DMD models, yet because exercise was induced before imaging scans, acute effects of exercise might confound our imaging findings. We believe, however, that at the time of scanning, which was typically 30 minutes after exercise, the effects of exercise would be limited. In addition, since each animal was serially studied using the same protocol, with the results of each animal being normalized with respect to its own baseline, the residual effects of exercise would be factored out in inter- and intra-animal comparisons.

## Conclusions

In summary, the present study clearly demonstrates that non-invasive imaging of biomarkers accurately reflects the disease state (ie., mdx mice are intermediate in disease severity to healthy wild-type mice and severely-affected udx mice). Furthermore, imaging of biomarkers allows us to delineate changes in muscle regeneration/degeneration beyond that achievable with traditional histological analyses of DMD, ie., changes in blood flow, blood volume and glucose metabolism *between *non-exercised and exercised groups of mdx mice. This could have enormous implications for much needed studies examining the mechanisms of DMD pathology that may improve or worsen as a result of exercise. Importantly, these technologies can potentially be translated to the clinic as an alternative to invasive procedures. Furthermore, our findings may now serve as a baseline for comparison with cell and gene therapeutics aimed at improving the quality of life for patients, and/or finding a cure for those affected with DMD or other neuromuscular diseases.

## Appendix

### Tracer Kinetic Modeling

Muscle blood flow (perfusion) and blood volume maps were calculated by means of the tracer kinetic model first described by Johnson and Wilson [48]. St Lawrence and Lee derived the adiabatic approximation solution which simplifies the calculation of perfusion and other related functional parameters in tissue such that it is independent of the relative magnitude of the permeability surface area (PS) product of the capillary endothelium to contrast agent to perfusion [22]. Since capillaries in muscle are permeable to contrast, the model divides muscle into two principal spaces; the intravascular space (IVS) and the extra-vascular space (EVS), which are separated by the permeable capillary endothelium [48]. The model uses three basic assumptions to arrive at a solution. First, the permeable capillary endothelium allows bidirectional diffusion of contrast agent between EVS and IVS. Second, in the capillaries there is an axial concentration gradient of contrast but the radial concentration gradient is assumed to be negligible. Third, within the EVS the tracer concentration is assumed to have a homogeneous spatial distribution (i.e. EVS is a compartment). The adiabatic approximation, as discussed by St. Lawrence and Lee [22]. assumes that the EVS contrast concentration is changing slowly (i.e. in a quasi-steady state) relative to the rate of change of concentration in the IVS (capillaries). Using the adiabatic approximation, the impulse residue function [22], H (t), can be represented as:(1)

where *T _c _*is the capillary transit time and F is blood flow so that blood volume is F*T_c _*according to the Central Volume Principle [49], E is the extraction fraction of contrast and *V_e _*is the distribution volume of contrast in the EVS. E relates to the PS of muscle capillaries via the following relationship [50]:(2)

If the concentration (enhancement) of contrast (arterial) input to the muscle, C_a_(t), is known, then the measured muscle contrast enhancement curve, Q (t), can be calculated as the convolution of C_a_(t) and H (t):(3)

where * is the convolution operator. The validity of equation 3 assumes that muscle blood flow is constant and Q (t) is linear with respect to C_a_(t).

The lower limbs are supplied by two common iliac arteries, C_a_(t) was obtained from the DCE-CT images with a ROI placed in one of the those arteries. From each 2 × 2 pixel block in the DCE-CT images, the muscle contrast enhancement curves, Q(t), was also obtained. Deconvolution between C_a_(t) and Q(t) with the CT Perfusion software (GE Healthcare) gave H(t), from the latter muscle blood flow (perfusion) and blood volume for the pixel block in question were derived with Eqs (1)-(3). Functional maps of muscle perfusion and blood volume were generated by repeating the deconvolution process for each pixel block.

## List of Abbreviations

ANOVA: analysis of variance; BF: blood flow; BV: blood volume; CT: computed tomography; DCE-CT: dynamic contrast-enhanced computed tomography; DGC: dystrophin-glycoprotein complex; DMD: Duchenne muscular dystrophy; Ex: exercised; FDG: fluorodeoxyglucose; GMs: gastrocnemius muscles; H&E: haematoxylin and eosin; HL: hind limb; Mdx: muscular dystrophy on the × chromosome; MR: magnetic resonance; NBF, NBV, NSUV: normalized blood flow, normalized blood volume, normalized standard uptake value; N.E.: non-exercised; nNOS: neuronal nitric oxide synthase; PCR: polymerase chain reaction; PET: positron emission tomography; ROI: region of interest; SUV: standard uptake value; Udx: mdx: utrn -/-; utrn: utrophin; W.t.: wild-type

## Competing interests

The authors declare that they have no competing interests.

## Authors' contributions

NA made substantial contributions to the acquisition of DCE-CT, PET-FDG and histological data, and data analysis and interpretation of DCE-CT and PET-FDG findings. IW made substantial contributions to the analysis of histological data, and to revision of the manuscript. RG generously supplied us with the mdx:utrn+/- breeding colony, and as an expert in muscle physiology and function, aided us in the experimental design of the mouse exercise regime and in interpretation of resulting findings. JH made substantial contributions to the acquisition of all data, placing tail catheters and aiding NA with all animal work and care. SD and DH were heavily involved in critically revising the manuscript for important intellectual content. TYL and LH made substantial contributions to the conception and design of the study, to the interpretation of data, and were heavily involved in critically revising the manuscript for important intellectual content. LH also contributed substantially to the acquisition of data, and is the corresponding author.

All authors have read and approved the final manuscript.

## Pre-publication history

The pre-publication history for this paper can be accessed here:

http://www.biomedcentral.com/1471-2474/12/127/prepub
